# The Impact of T Cell Vaccination in Alleviating and Regulating Systemic Lupus Erythematosus Manifestation

**DOI:** 10.1155/2016/5183686

**Published:** 2016-12-04

**Authors:** Liuye Huang, Yuan Yang, Yu Kuang, Dapeng Wei, Wanyi Li, Qin Yin, Juan Pang, Zhongwei Zhang

**Affiliations:** ^1^Department of Microbiology, West China School of Preclinical and Forensic Medicine, Sichuan University, Chengdu, China; ^2^West China School of Public Health, No. 4 West China Teaching Hospital, Sichuan University, Chengdu, China; ^3^Department of Rheumatology, Jinjiang District People's Hospital of Chengdu City, Chengdu, China; ^4^ICU, West China Hospital, Sichuan University, Chengdu, China

## Abstract

*Objective*. Systemic lupus erythematosus (SLE) is an autoimmune disease identified by a plethora of production of autoantibodies. Autoreactive T cells may play an important role in the process. Attenuated T cell vaccination (TCV) has proven to benefit some autoimmune diseases by deleting or suppressing pathogenic T cells. However, clinical evidence for TCV in SLE is still limited. Therefore, this self-controlled study concentrates on the clinical effects of TCV on SLE patients.* Methods*. 16 patients were enrolled in the study; they accepted TCV regularly. SLEDAI, clinical symptoms, blood parameters including complements 3 and 4 levels, ANA, and anti-ds-DNA antibodies were tested. In addition, the side effects and drug usage were observed during the patients' treatment and follow-up.* Results*. Remissions in clinical symptoms such as facial rash, vasculitis, and proteinuria were noted in most patients. There are also evident reductions in SLEDAI, anti-ds-DNA antibodies, and GC dose and increases in C3 and C4 levels, with no pathogenic side effects during treatment and follow-up.* Conclusions*. T cell vaccination is helpful in alleviating and regulating systemic lupus erythematosus manifestation.

## 1. Introduction

Systemic lupus erythematosus (SLE, lupus) is a chronic autoimmune disease that is characterized by diffuse immune inflammation damage on connective tissue. Lupus occurs ten times more often in women compared to men [1], and annual direct medical costs of adult patients with active SLE are very high [2, 3]. Currently, antimalarial, steroids, and immunosuppressive drugs are three fundamental therapies for SLE, with the aim of reducing the severity of exacerbation and prolonging the periods between successive episodes. However, not only do these drugs not fit well with all the situations in SLE, but also unavoidable side effects may occur, such as complications, irreversible organ damage, and, with long-term and high-does use, a decreased quality of life [4]. Activated autoreactive T cells that assist B cells overreaction and overproduction of autoantibodies are thought to be crucial for the pathogenesis of autoimmune diseases, including SLE. Biopharmaceuticals, including inhibitors for B cells, T cells, costimulating molecules, complement activation, and cytokine, are promising target therapies in SLE due to the reason that they inhibit the proliferation of autoantibodies or regulate the abnormal immune system [4]. However, most of these biopharmaceuticals are in clinical trials and are not currently available [5]. Besides, few of them have achieved a satisfactory therapeutic effect in clinical tests of SLE. For example, abatacept and rituximab (RTX), which were highly anticipated in treating SLE, due to their excellent performance in treating other autoimmune diseases, led to unpromising results in SLE clinical trials [6–9]. Even belimumab, the first biologic to be approved by US FDA and European Medicines Agency (EMA), only showed limited clinical effects among mild to moderate patients, and a great percentage of the patients did not respond to it [10–12]. Furthermore, these drugs are often costly and required long-term use which makes them inaccessible for most SLE patients. For example, the cost of belimumab is prohibitive for most patients and it requires 52 weeks of therapy to achieve its ideal clinical effect [10, 11]. Therefore, there is a considerable need for more effective and safe therapies for SLE patients.

Some former research has suggested that motivating autoreactive T cells may break the balance of the regulatory immune system so as to cause autoimmune diseases (ADs) [13]. Attenuated T cell vaccination as a novel therapy was first raised in 1981 [14]. It is believed that TCV is critical in regulating the autoimmune system and alleviating clinical manifestations of ADs, because it particularly attenuates pathogenic autoreactive T cells. And, indeed, more and more research demonstrates the beneficial effects of TCV in the treatment of ADs, not just in various animal models or clinical trials, including SLE [13].

Remarkably, in 2005, the attenuated autologous T cells were first tested in six SLE patients with mild lupus manifestations, and clinical improvements of patients including reduced SLEDAI scores and autoantibodies parameters, as well as relieved clinical features such as facial rash, were observed after four times of injections of inactivated CD4+ T cells [15]. In this study, we started with this idea and took it further with 16 SLE patients to demonstrate the effects of TCV in alleviating and regulating SLE.

## 2. Patients and Methods

### 2.1. Patients

Patients were enrolled who had a diagnosis of SLE according to the 1997 ACR classification criteria for more than half a year and had a positive antinuclear antibody. Patients were excluded for the following reasons: if they had severe SLE activity demonstrated by the SLEDAI score at screening (SLEDAI ≥ 15); if they suffered from important organ diseases including heart, kidney, lung, and nerve, such as central nervous system lupus and acute glomerulonephritis; if they had serious current or recent infection; if they were pregnant, lactating, or planning to be pregnant within the next year; or if they had previously received biological treatment.

As a result, sixteen patients (fifteen women and one man) were included. Their age ranged from 13 to 52, on average 27.19 ± 11.26 years; their mean disease duration was 6.97 ± 4.73 years, ranging from 0.5 to 14 years. They experienced mild to moderate lupus manifestations and all in disease active stage. Among them, twelve patients underwent low complementemia, eight facial rash, seven arthralgia or myalgia, six continuous proteinuria, three positive ds-DNA, two Raynaud's phenomena, and three abnormalities of peripheral blood counts (P10, leukocyte < 4 × 10^9^/L; P8 and P12, platelet < 100 × 10^9^/L); there were no patients with fever. Before TCV, all patients were treated with glucocorticoid for at least half a year and have recently received stable dosage for more than three months. Some of them also received hydroxychloroquine (HCQ) and immunosuppressive drugs and have been supported by infrared therapy selectively during their past treatment, however with no visible improvement in their clinical manifestation or accompanied with severe relapsing.

The study obtained approval by Medical Ethics Committee of Sichuan University (JJ2014003). All patients provided written informed consent prior to vaccination. Patients who were under 18 years of age gained consent from their legal guardians.

#### 2.1.1. Preparation of T Cell

The approaches for T cell discretion and expansion were similar to previous studies [15–18]. In brief, peripheral blood mononuclear cells (PBMC) were taken from every patient particularly by Ficoll density gradient centrifugation. Subsequently, part of the PBMC were irradiated as feeder cells for the proliferation of T cells. The live PBMC near 3 × 10^5^ per well were cultured with the irradiated ones at the density ratio of about 10 : 1 in the 6-well plates. Cells were fed in RPMI 1640 (Gibco, Grand Island, NY) medium, supplemented with rhIL-2 (50 IU/mL) and 10% heat-inactivated autologous serum of every patient, respectively. The culture medium was changed every three to four days. Seven to ten days later, the cells were up to 10^6^ in total and further selected with anti-CD4 to isolate the CD4+ cells by magnetic microbeads according to the protocol (MACS, Miltenyi Biotech). The isolated T cells were collected, further expanded, and stimulated in the T75 flasks by adding phytohemagglutinin 2 *μ*g/mL, rhIL-2 100 IU/mL, and 10% heat-inactivated autologous serum. The cell numbers in this stage vary from 10^7^ to 10^8^ in total, on average 2 × 10^7^ among different patients. After the proliferation and selection, T cells were attenuated by 7000 rad *γ* irradiation and collected distinctively. A portion of the cells was first packed in brown glass, about 5 × 10^6^ cells per phial, and stored in 4°C for the first injection; a small part of the cells were kept for the following examinations, and the remaining cells were frozen in liquid nitrogen for future injection.

For understanding the changes of the antiautoreactive T cell antibodies in vivo after vaccine, the patients' peripheral serum in different stages of vaccination was obtained, including pre-TCV and the fourth week (W4) and the eighth week of vaccination (W8), as the first antibody, to react with the inactivated sensitized T cells, which were previously prepared. The nuclei of these cells were colored blue by DAPI, while the secondary antibody was goat anti-human IgG antibody, FITC conjugate (Thermo fisher, Massachusetts) for green coloring, which would mark the reaction between the first antibody and the antigen.

### 2.2. Therapeutic Scheme

#### 2.2.1. General Drugs for SLE Patients

All patients received the glucocorticoid (GC, 5–60 mg/d), hydroxychloroquine (HCQ, 0–0.4 g/d), and immunosuppressive drugs: methotrexate (MTX), mycophenolate mofetil (MMF), or leflunomide (LEF) in the same dosage as that used before, and the dosage was adjusted based on their prognosis.

#### 2.2.2. T Cell Vaccination

Each patient was vaccinated by a single unit of vaccine (about 5 × 10^6^ cells) every time, with bum intramuscular injection, and was revaccinated in the second, fourth, and the eighth week of vaccination.

#### 2.2.3. Supporting Drugs

All patients received supporting drugs during the treatment, including tablets of folic acid (4 mg/d), vitamin B (20 mg/d), calcium (varying with the age of every patient), and H2 receptor blocker, such as lansoprazole and omeprazole (15–30 mg/d). Furosemide and potassium chloride were provided for patients with edema.

#### 2.2.4. Supporting Apparatus

Infrared therapy apparatus was used for patients with arthralgia or myalgia, keeping the same treatment protocol as before, varying from once a week to once a month. There are three kinds of infrared lights based on their wavelength: the near-, mid-, and far-infrared. Among them, the near-infrared (wavelength 700–1400 nm) kind functions best in penetrating tissues and warming blood vessels and thus causes muscle relaxation and telangiectasia, increasing peripheral bloodstream to relieve cardiovascular system related diseases and musculoskeletal pain; and the far-infrared kind is also believed to exert clinical effects [19]. Furthermore, infrared therapy has been proved to be effective in relieving back and musculoskeletal pain in autoimmune diseases such as rheumatoid arthritis [20].

### 2.3. Analysis and Evaluation

#### 2.3.1. Safety and Efficiency Evaluation

Routine blood, blood biochemistry, and immune indexes tests were performed before every vaccination. Patients were allowed to leave in case of no discomfort in their sitting and peaceful state for 30 minutes after vaccination. Patients were followed up every four weeks when they were of low complementemia (LC) but every twelve weeks when their complements parameters were normal, with clinical manifestation, adverse events, urinary protein level, complements, and antibodies being observed, as well as adjusting the usage of their general drugs and supporting therapies.

*SLE Disease Activity Index (SLEDAI)*. The patients' disease activity in different status was measured by a trained physician, who was totally blind to this study. The grades were based on the SLEDAI-2K, which was a major scoring system to evaluate the activity of lupus [21].

#### 2.3.2. Study Duration

The 16 patients were enrolled between February 6, 2015, and September 1, 2015, so, until the end of the year 2015, the latest follow-up weeks ranged from W14 to W40 among different patients—on average 27 weeks.

#### 2.3.3. Statistical Methods

Descriptive statistics were adopted in noting the changes of clinic manifestation and antibodies; paired Student's *t*-test was applied to examine the changes in SLEDAI, complements 3 (C3) and 4 (C4) parameters, and routine dosage of GC. All the statistics were carried out on SPSS 17.0 software. It was considered as statistically significant when *P* value was less than 0.05 and marked with *∗*.

## 3. Results

### 3.1. The Changes in Clinical Manifestation

During the TCV treatment and follow-up, most patients experienced a reduction in SLEDAI and an improvement in clinical features, including the constant remission of skin rash, ulcer, vasculitis, low complementemia, proteinuria, alopecia, fatigue, and Raynaud's phenomena. In addition, no relapse was experienced. Specific cutaneous lesions: the facial rash of eight patients before TCV attenuated in W4 mostly and vanished at last, with no new generation and relapsing during treatment and follow-up, including the facial and shoulder-back herpes of P1, as well as the intractable facial rash of P14, which has relapsed again and again during the past fourteen years. However, P4 and P8 have experienced facial rash relapse in their W28 and W21, respectively; the facial rash of P4 happened after physical activity and that of P8 happened after inappropriate use of make-up, and both of them recovered soon by giving GC.

Nonspecific cutaneous lesions: P3 and P16 were vasculitis, manifested in severe ulcer of fingertips and bilateral metacarpophalangeal joints separately, and recovered gradually during vaccination. P3 has suffered from Raynaud's phenomena in the winter for the past years but with no relapsing during the winter of this year. P14 also recovered from Raynaud's phenomena with no relapsing during her treatment and follow-up.

Seven patients with arthralgia or myalgia recovered in W4 with no relapse during vaccination and follow-up. For the specific changes of clinical manifestations and SLEDAI scores in patients with TCV please see Table 1.

Among the 16 patients, P5, P6, P9, P11, P12, and P13 suffered from continual proteinuria before vaccination but have been improved since the TCV treatment, as Table 2 shows.

### 3.2. The Changes in C3 and C4 Parameters of Patients

Twelve patients underwent low complementemia before first vaccination, but, in the fourth week, for eleven of them, the C3 and C4 level raised during the treatment or in the follow-up (W4: C3, *P* = 0.038^*∗*^; C4, *P* = 0.017^*∗*^) (see Table 3). In the latest follow-up week, all the patients have recovered from the low complementemia, except P8, who was still in the low C3 parameter.

### 3.3. The Changes in Antibodies of Patients

All the patients remained ANA positive during the study. Among them, P16's ANA changed from 1 : 320 speckled pattern and 1 : 100 homogeneous pattern into 1 : 320 speckled pattern. The anti-ds-DNA antibodies of three patients (P4, P7, and P11) have turned negative in W8.

### 3.4. The Changes in Routine Dosage of GC

**Figure 1:**
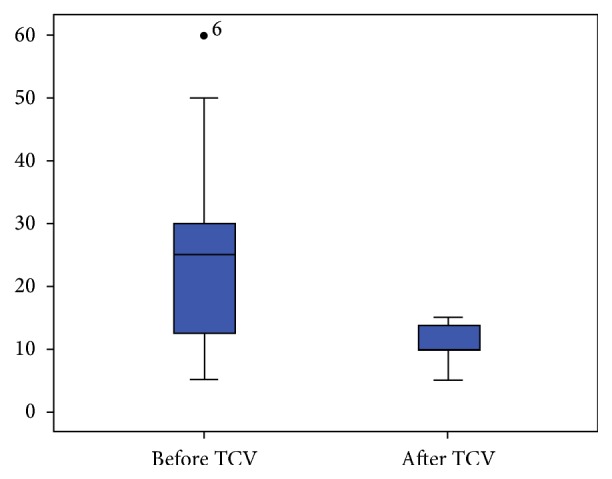
The changes of routine GC dosage.

TCV has decreased the routine dosage of GC significantly. The mean GC before TCV was 25.31 ± 3.83 mg/d, and it has become 10.78 ± 0.78 mg/d after TCV treatment (*P* = 0.001^*∗*^, 95% CI: 7.313, 21.750). P6 experienced the highest dose of GC (60 mg/d) as one of her usual drugs, but it reduced to 15 mg/d after the treatment. However, P8, who used GC in 5 mg/d before TCV, has changed into using 10 mg/d because of her relapsing rash in W21. For the changes of routine GC dosage see Figure 1.

### 3.5. The Changes in Antiautoreactive T Cell Antibodies of Periphery Blood

**Figure 2:**
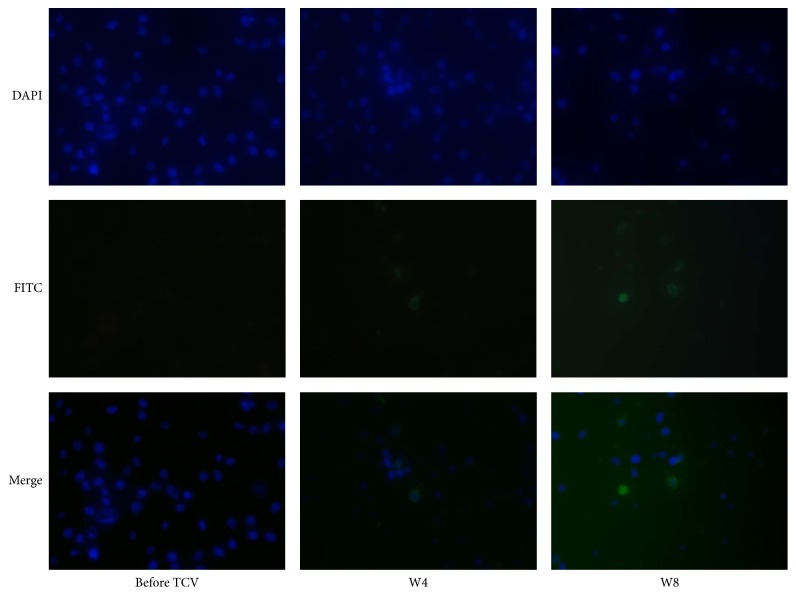
The changes in antiautoreactive T cell antibodies of periphery blood.

As the pictures in Figure 2 show, FITC colored antiautoreactive T cell antibodies (IgG) were observed in W4 and enhanced in W8. The magnification of the immunofluorescence is 200x.

### 3.6. Side Effects

During the TCV treatment and follow-up period, two of the patients experienced low fever and mild gastrointestinal upset while T cells were injected but recovered within 30 minutes; the others underwent no significant side effects manifestation, such as flu-like symptom and urticaria.

## 4. Discussion

SLE has been known for its damage to multiple systems and organs, relating to the accumulation of immune complex (IC) induced type III allergy generally. The overgenerated autoantibodies are believed to assist the formation of the immune complex (IC). During the pathogenesis of SLE, autoreactive T cells stimulate B cells to differentiate, proliferate, mature, and switch classes to support the production of autoantibodies [22, 23].

Attenuated T cells are believed to alleviate manifestation of autoimmune diseases through clearing pathogenic autoreactive T cells [13, 18]. The study supported this idea because antiautoreactive T cell antibodies were observed in W4 and increased in W8, which concurred with the fact that most patients' clinical manifestations were relieved in W4 and were evidently improved in W8, including decreasing SLEDAI and remissions on various clinical features, such as facial rash, low complementemia, and proteinuria.

Anti-idiotypic and antiergotypic networks are possible explanations for the mechanism of TCV. In brief, the anti-idiotypic network which consists of CD8+ and CD4+ anti-idiotypic T cells is a distinctive immune response of TCV. The CD8+ T cells deplete or suppress pathogenic T cells by inducing cytotoxicity to inhibit CD4+ effector T cells, while the CD4+ T cells produce cytokines (IL-4, IL-10) to promote regulatory immune balance. Both of the T cells require the help of APC. Besides, the reactive B cells may make a marginal contribution to the deletion or suppression of autoreactive T cells by providing anti-idiotypic antibodies. Antiergotypic T cells, one of the main composition elements of the antiergotypic network, were selectively activated as a result of TCV and secrete cytokines including IL-10 or TGF-*β* to downregulate the autoreactive T cells and thus alleviate the disease severity [13, 22].

Furthermore, the evident improvement on C3 and C4 levels of patients was observed. Generally, complement is crucial for stopping the formation of IC [24, 25], as well as dissolving the formed and deposited IC. The low C3 and C4 levels may result in a vicious cycle, because, in turn, accumulated IC activates and consumes complement through classical pathway [26]. Besides, autoantibodies such as anti-C1q could damage complement [27], and it has recently been reported that anti-C3 autoantibody levels correlated with disease activity [28]. Most SLE patients are complement deficient, and their immune system cannot clear IC effectively, increasing IC deposits to cause damage to organs or systems. So, in this study, the constant remissions in low complementemia with no relapsing of patients during treatment and follow-up may be attributed to the clearance of autoantibodies by injecting attenuated whole T cells and prevent the formation and deposition of IC in vessels, kidneys, and articulation, thus relieving the clinical symptoms of multiple systems and organ damage in vivo.

GC has been an essential therapy for SLE because it works effectively in anti-inflammatory and antiallergic reactions, as well as assisting in improving SLE response to immunosuppressive therapy [29]. However, an increasing number of reports have demonstrated glucocorticoid-induced damage, such as osteoporosis, diabetes, and hypertension [4, 12, 30, 31] due to long-term and high-dose use in the treatment of SLE. Patients with high prednisone (>30 mg/day) were more likely to accrue new damage, while low-moderate doses (≤30 mg/day) are similarly more effective and safer for treating active lupus [32]. In this study, vaccination with attenuated T cells was found to be helpful in lowering the GC doses of patients regular usage, thus preventing patients from GC related damage. In addition, higher GC use was related to more health care utilization and costs [33], so TCV may contribute to the lower cost on SLE through reducing routine GC dosage.

No apparent side effects were observed during the treatment and follow-up period, so we assumed attenuated T cell vaccination is safe in use for SLE, which accords with previous studies [15, 17, 18, 34].

This quested clinical study aimed at exploring the effects of TCV on SLE patients. GC, immunosuppressive drugs, and HCQ are still mainstay treatments for SLE currently [35]. In this study, most patients cannot completely refrain from the main drugs so we set it as a self-controlled study to demonstrate the clinical efficacy of TCV on SLE patients through contrasting the differences in clinical manifestation, the blood parameter, and drugs before and after the treatment. Given the premise that all the patients have been diagnosed with SLE for more than half a year and stable treatment schemes which involved GC, HCQ, and immunosuppressive drugs have been used on them for more than three months, however, patients' clinical and experimental indices were not improved or accompanied with severe relapse. At the beginning of the study, patients' treatment scheme and drug usage remain exactly the same as their former ones. However, during the T cell treatment and follow-up study, not only were patients' clinical symptoms relieved, but the average dosage of GC decreased evidently. Furthermore, the increasing antiautoreactive T cell antibodies in periphery blood in different weeks accord with such improvement among patients. Therefore, it can be inferred that the TCV benefits patients with SLE. But it still remains an open ending, which means randomized control studies with a large population are required in the future to illustrate the exact effect of TCV on SLE patients.

Currently, although former research of TCV has promised bright future in the treatment of ADs, implementation of TCV in SLE still faces great challenges. Not only are the mechanisms waiting to be interpreted through animal experiments, but also the proper treatment scheme, referring to vaccination cells, dosage, manner, and period in clinical practice due to the lack of relevant clinical studies, still remains unclear. Furthermore, each T cell vaccine is generated from every single patient distinctively; this can be an advantage or disadvantage, for, on one hand, it may hinge the extensive commercial manufacture, but, on the other hand, it highlights the idea of personalized medicine, which may benefit SLE patients more precisely. Besides, similar to any other new therapy, the health beneficial cost of TCV has to be carefully taken into account when referring to choosing the “best” treatment for patients: it is inevitable that TCV cost more in production compared to classical drugs; however, TCV works effectively in lowering medical cost by decreasing the GC usage [33] and alleviating clinical manifestation [36]. Because these questions remain to be clarified, clinical trials with a large population and animal tests for interpreting mechanisms are all necessary for advancing the utilization of TCV in SLE.

To summarize, TCV was associated with remissions in clinical symptoms and reductions in SLEDAI and anti-ds-DNA antibodies and GC doses and increases in C3 and C4 levels, with no pathogenic side effects during treatment and follow-up, which may prove TCV functions effectively and safely in alleviating and regulating the manifestation of SLE patients.

## Figures and Tables

**Table 1:** Changes in clinical manifestations and SLEDAI scores of patients with TCV.

Patients	Before TCV		W4		W8		The latest follow-up week	
	Clinical features	SLEDAI	Clinical features	SLEDAI	Clinical features	SLEDAI	Clinical features	SLEDAI
P1	Facial and shoulder-back herpes, LC	4	Facial and shoulder-back herpes, LC	2	Facial rash	0	None	0
P2	Facial rash, alopecia, LC	6	Alopecia	2	None	0	None	0
P3	Arthralgia and myalgia, severe ulcer on finger tips, alopecia, LC, relapsing Raynaud's phenomena in every winter	12	Alopecia, ulcer on finger tips	10	Alopecia	2	No Raynaud's phenomena this winter	0
P4	Arthralgia, alopecia, LC	6	Alopecia	2	Alopecia, LC	4	None	0
P5	LC, proteinuria	2	LC, proteinuria	2	Proteinuria	0	None	0
P6	Arthralgia, edema, proteinuria, urinary occult blood, LC	10	Proteinuria, LC	6	Urinary occult blood, proteinuria	8	Proteinuria	4
P7	Facial rash, alopecia	6	Alopecia	2	None	0	None	0
P8	LC	5	—	—	LC	2	LC	2
P9	Alopecia, fatigue, myalgia, proteinuria, urinary occult blood, LC	12	Alopecia, proteinuria, LC	8	Alopecia, proteinuria, LC	8	Alopecia, proteinuria	2
P10	Facial rash, arthralgia and myalgia, LC	5	LC	2	LC	2	None	0
P11	Alopecia, arthralgia and myalgia, facial rash, fatigue, lips cyanosis, dyspnea, proteinuria	10	Alopecia, lips cyanosis, dyspnea, proteinuria	2	Alopecia	2	Alopecia	2
P12	Arthralgia, alopecia, edema, fatigue, LC, proteinuria	9	Alopecia, edema LC, proteinuria	8	Alopecia, edema, proteinuria	6	Proteinuria	4
P13	Facial rash, proteinuria, LC	8	Facial rash, proteinuria, LC	6	Facial rash, proteinuria	4	None	0
P14	Facial rash, Raynaud's phenomena	2	Facial rash, Raynaud's phenomena	0	Facial rash, Raynaud's phenomena	0	None	0
P15	Arthralgia, alopecia, LC	4	Alopecia, LC	4	Alopecia	2	None	0
P16	Ulcer of skin on bilateral metacarpophalangeal joints, facial rash, dry mouth and eyes	10	Facial rash, ulcer on knuckle	8	Facial rash, ulcer on knuckle	8	None	0

*P* value				0.000^*∗*^		0.000^*∗*^		0.000^*∗*^

“—” means data absence.

**Table 2:** The changes in urinary protein and occult blood.

Patients	Before TCV	W4	W8	The latest follow-up week
P5	1 + 0.3 g	1 + 0.3 g	1 + 0.3 g	None
P6	3 + 3.0 g, urinary occult blood 3+	2 + 1.0 g	2 + 1.0 g, urinary occult blood 2+	2 + 1.0 g
P9	2 + 1.0 g, urinary occult blood 3+	2 + 1.0 g	2 + 1.0 g	1 + 0.3 g
P11	1 + 0.3 g	1 + 0.3 g	None	None
P12	3 + 3.0 g	2 + 1.0 g	2 + 1.0 g	2 + 1.0 g
P13	3 + 3.0 g	2 + 1.0 g	2 + 1.0 g	1 + 0.3 g

**Table 3:** The changes in C3 and C4 parameters of patients.

Patients	C3 (0.9–1.8 g/L)			C4 (0.1–0.4 g/L)		
	Before TCV	W4	W8	Before TCV	W4	W8
P1	0.73↓	0.68↓	0.98	0.12	0.11	0.14
P2	0.64↓	0.95	0.93	0.12	0.21	0.27
P3	0.65↓	1.3	1.09	0.05↓	0.25	0.17
P4	0.56↓	0.98	0.89↓	0.06↓	0.11	0.12
P5	1.00	0.91	1.01	0.03↓	0.09↓	0.13
P6	0.38↓	0.80↓	0.9	0.05↓	0.16	0.15
P7	1.08	1.04	1.24	0.14	0.18	0.27
P8	0.85↓	—	0.69↓	0.19	—	0.14
P9	0.64↓	0.59↓	0.62↓	0.07↓	0.11	0.12
P10	0.62↓	0.50↓	0.61↓	0.09↓	0.02↓	0.11
P11	1.02	—	1.05	0.16	—	0.19
P12	0.64↓	0.89↓	1.19	0.04↓	0.13	0.21
P13	0.58↓	0.66↓	0.91	0.07↓	0.09↓	0.11
P14	1.20	—	1.27	0.25	—	0.32
P15	0.58↓	0.84↓	0.79	0.16	0.19	0.16
P16	1.43	—	1.38	0.42↑	—	0.48

*P* value		0.038^*∗*^	0.004^*∗*^		0.017^*∗*^	0.001^*∗*^

“—” means data absence.

## Key Defined Acronyms

TCV: Attenuated T cell vaccination
SLE: Systemic lupus erythematosus
SLEDAI: Systemic lupus erythematosus disease activity index
C3: Complement 3
C4: Complement 4
PBMC: Peripheral blood mononuclear cells
AD(s): Autoimmune disease(s)
GC: Glucocorticoid
HCQ: Hydroxychloroquine
MTX: Methotrexate
MMF: Mycophenolate mofetil
LEF: Leflunomide
LC: Low complementemia.
